# Glial overexpression of carbonic anhydrase *cah-4* promotes glial and systemic autophagy, and reduces polyglutamine aggregation in *C. elegans*

**DOI:** 10.17912/micropub.biology.002259

**Published:** 2026-07-12

**Authors:** Lei Wang, Laura Bianchi

**Affiliations:** 1 Department of Physiology and Biophysics, Miller School of Medicine, University of Miami, 1600 NW 10th Ave, Miami, FL, 33136; 2 Department of Physiology and Biophysics, University of Miami

## Abstract

Glia regulate neuronal function and organismal physiology, but how glial mechanisms control proteostasis across tissues remains incompletely understood. We showed that alkalinization of Amphid sheath (AMsh) glia, achieved by loss of the chloride channel *clh-1* or overexpression of the carbonic anhydrase *cah-4* , promotes longevity and stress resistance. While these studies established a role for glial pH in aging, the effects of *cah-4* overexpression on autophagy and proteostasis were not examined. Here, we show that *cah-4* overexpression in AMsh glia increases autophagy locally and systemically, and reduces neuronal polyglutamine aggregation. These data confirm that glial alkalinization promotes local and systemic proteostasis.

**Figure 1. cah-4 overexpression increases autophagy and reduces proteotoxic stress:** (A) Quantification of the number of LGG-1 puncta (autophagosomes) in AMsh glia of WT and *cah-4* overexpression nematodes, indicating autophagosome abundance at the cellular level. n = 33 and 36 AMsh glial cells, respectively.(B) Quantification of LGG-1 fluorescence intensity in AMsh glia of WT and *cah-4* overexpression nematodes, reflecting overall LGG-1 levels in these cells. Fluorescence values were normalized to WT. n = 33 and 36 AMsh glial cells, respectively.(C) Quantification of the number of LGG-1 puncta (autophagosomes) across the whole animal in WT and *cah-4* overexpression nematodes, indicating global autophagy levels. n = 23 and 26 worms, respectively.(D) Quantification of LGG-1 fluorescence intensity in the whole animal in WT and *cah-4* overexpression nematodes. Fluorescence values were normalized to WT. n = 23 and 26 worms, respectively.(E) Quantification of polyQ aggregates in WT and *cah-4* overexpression nematodes at day 1 of adulthood. n = 28 worms per genotype.(F) Same as in E for day 5 of adulthood. n = 29 and 30 worms, respectively. Data are expressed as individual data points and mean ± SE. Statistics were performed using an unpaired Student's t-test. * and **** indicate p < 0.05 and 0.0001, respectively.

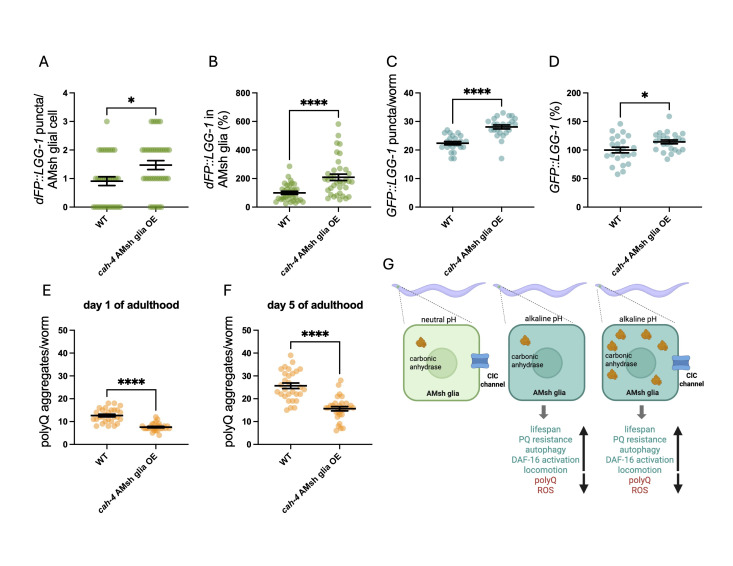



## Description

Glial cells account for approximately half of the cells in the nervous system and regulate neuronal output and synaptic plasticity, while also providing metabolic support to neurons (Demmings et al., 2025; Sancho et al., 2021). During aging, glia undergo functional changes that shift them from protective to dysfunctional states, contributing to neuronal decline and disease (Saijo & Glass, 2011; Salminen et al., 2011). However, the extent to which glial dysfunction or modulation actively drives organismal aging remains unclear.

&nbsp;

Studies in *Caenorhabditis elegans* have begun to address this question, demonstrating that activation of stress response pathways in glia can influence organism-wide physiology through non-cell-autonomous signaling. For example, activation of UPR _ER_ , UPR _MT_ , or heat shock responses in CEPsh glia extends lifespan and reduces protein aggregation through signaling to distal tissues (Bar-Ziv et al., 2023; Frakes et al., 2020; Gildea et al., 2022). These findings indicate that glia can coordinate systemic responses to stress.

&nbsp;

One core function of glia is the regulation of ionic homeostasis. In *C. elegans* , the Amphid sheath (AMsh) glia control the ionic environment surrounding sensory neurons, including bicarbonate and chloride levels (Fernandez-Abascal & Bianchi, 2022; Fernandez-Abascal et al., 2022; Grant et al., 2015; Han et al., 2013; Wang et al., 2008; Wang et al., 2012). We previously showed that the chloride channel *clh-1* regulates intracellular pH (pH _i_ ) in these cells, and that loss of *clh-1* leads to AMsh glial alkalinization (Fernandez-Abascal & Bianchi, 2022; Grant et al., 2015). We also demonstrated that loss of *clh-1* promotes lifespan extension, oxidative stress resistance, and reduced polyglutamine (polyQ) aggregation, and is accompanied by activation of protective pathways, including autophagy, via activation of the DAF-16 /FoxO transcription factor (Wang et al., 2025). We further showed that these effects depend on glial pH, as they are reversed by knockdown of pH regulators such as *abts-3* or *cah-4* . Importantly, overexpression of the carbonic anhydrase *cah-4* in AMsh glia is sufficient to alkalinize these cells and to recapitulate key organismal phenotypes of *clh-1* loss, including increased lifespan, reduced reactive oxygen species, enhanced paraquat resistance, and increased DAF-16 nuclear localization (Wang et al., 2025). However, despite these parallels, the effects of *cah-4* overexpression on autophagy and proteostasis were not examined.

&nbsp;

Here, we asked whether *cah-4* overexpression is sufficient to induce autophagy. Using LGG-1 reporters, we found that animals overexpressing *cah-4* in AMsh glia display increased LGG-1 fluorescence and a higher number of fluorescent puncta in these cells, consistent with elevated autophagosome formation. In addition, using a whole-body *lgg-1p::gfp:: lgg-1* reporter, we observed increased autophagy across the organism. These findings mirror the local and systemic activation of autophagy previously observed in *clh-1* mutants. We next assessed whether *cah-4* overexpression affects proteostasis. In animals expressing neuronal polyQ (Q67), overexpression of *cah-4* in AMsh glia resulted in reduced aggregate accumulation at day 1 and day 5 of adulthood. This phenotype is consistent with the reduced polyQ aggregation previously reported in *clh-1* mutants (Wang et al., 2025).

&nbsp;

Together, these results extend our previous findings by showing that *cah-4* overexpression not only promotes longevity and stress resistance but also induces autophagy and improves proteostasis. These data support a model in which alkalinization of a small population of glial cells is sufficient to activate organism-wide protective responses.

## Methods

**Strains and maintenance**

*C. elegans* strains were maintained at 20 °C on nematode growth medium (NGM) plates seeded with E. coli OP50 (Brenner, 1974). Experiments were performed on synchronized hermaphrodites obtained by bleaching gravid adults. The wild-type strain was N2 Bristol. For autophagy assays in AMsh glia, animals expressing Cerulean-Venus(dFP):: LGG-1 were used. For global autophagy assays, animals expressing GFP:: LGG-1 under its endogenous promoter ( *lgg-1p::GFP:: lgg-1* ) were used (Kang et al., 2007; Melendez et al., 2003). For polyQ assays, animals expressing neuronal Q67::CFP were used (Brignull et al., 2006).

&nbsp;

**Molecular biology**

The *cah-4* overexpression construct was previously described (Wang et al., 2025). Briefly, an 843 bp cDNA corresponding to the *cah-4* isoform a ( *cah-4 a* ) was amplified from *C. elegans* cDNA using primers engineered to introduce KpnI and AvaI restriction sites at the 5′ and 3′ ends, respectively. The *cah-4 a* cDNA was then subcloned into the pPD95.75 vector downstream of the *spig-11* promoter (Purice et al., 2025), and the resulting construct was confirmed by restriction digestion and sequencing.

&nbsp;

**Fluorescence microscopy**

Fluorescence imaging was performed on synchronized animals at the indicated ages. Worms were immobilized on 2% agar pads (prepared in M9 buffer) using 300 mM sodium azide. Images were acquired with an EVOS FL Auto 2 imaging system using either a 20× objective for whole-animal imaging or a 100× objective for AMsh glial cell imaging. For whole-animal analyses, three to five 20× fields were stitched to reconstruct each animal prior to quantification. Image analysis was performed in Fiji (ImageJ). Fluorescence intensities were measured after background subtraction. For AMsh glial measurements, background was taken from an adjacent region within the worm body, whereas for whole-animal measurements, background was defined from the coverslip. Fluorescence values were normalized and expressed as a percentage of wild type within the same age group. Both fluorescence intensity and the number of fluorescent puncta per AMsh glial cell or per animal were quantified in a genotype-blinded manner.

&nbsp;

**Statistical analysis**

Statistics and graphical analysis were performed using Graph Pad Prism 10. Data are presented as mean ± SEM. Statistical comparisons were performed using unpaired Student's t-tests.

## Reagents

**Table:** 

Strain	Genotype	Source
N2	N2	CGC
DA2123	*adIs2122 [lgg-1p::GFP:: lgg-1 + rol-6 ( su1006 )] *	(Melendez et al., 2003)
BLC1128	* blcEx643[spig-11p:: cah-4 (cDNA);mec-4p::mcherry];blcEx630[(spig-11p::dFP:: lgg-1 ;mec-4p::mcherry] *	This study
BLC969	* blcEx630[spig-11p::dFP:: lgg-1 ;mec-4p::mcherry] *	This study
BLC1127	* blcEx643[spig-11p:: cah-4 (cDNA);mec-4p::mcherry]; adIs2122 [lgg-1p::GFP:: lgg-1 + * *rol-6 ( su1006 )]* &nbsp;	This study
AM44	*rmIs190[rgef-1p::Q67::CFP]*	(Brignull et al., 2006)
BLC1126	* blcEx643[spig-11p:: cah-4 (cDNA);mec-4p::mcherry]; rmIs190[rgef-1p::Q67::CFP] *	This study
